# Unveiling the Potential Prebiotic Effects of Edible Mushroom *Pleurotus djamor* During *In Vitro* Colonic Fermentation

**DOI:** 10.1021/acs.jafc.4c06620

**Published:** 2024-11-21

**Authors:** Giuliane Moura Andrade, Evandro Leite de Souza, Jhonatan Rafael Zárate-Salazar, Jordana Nunes de Oliveira, Josean Fechine Tavares, Marcos dos Santos Lima, Rossana Lucena de Medeiros, Thatyane Mariano Rodrigues de Albuquerque, Fillipe de Oliveira Pereira

**Affiliations:** † Postgraduate Program in Nutrition Sciences, Health Sciences Center, Federal University of Paraíba, João Pessoa 58051-900, Brazil; ‡ Department of Nutrition, Health Sciences Center, Federal University of Paraíba, João Pessoa 58051-900, Brazil; § Postgraduate Program in Ecology and Conservation, Federal University of Sergipe, São Cristóvão 49680-000, Brazil; ∥ Postgraduate Program in Natural and Synthetic Bioactive Products, Health Sciences Center, Federal University of Paraíba, João Pessoa 58051-900, Brazil; ⊥ Department of Food Technology, Federal Institute of Sertão Pernambucano, Petrolina 56302-100, Brazil; # Fungi Research Group, Academic Unit of Health, Education and Health Center, Federal University of Campina Grande, Cuité 58429-900, Brazil; ∇ Department of Nutrition, Health Sciences Center, Federal University of Paraíba, João Pessoa 58175-000, Brazil; ○ Fungi Research Group, Academic Unit of Health, Education and Health Center, Federal University of Campina Grande, Cuité 58175-000, Brazil

**Keywords:** edible mushroom, phenolic compounds, short-chain fatty acids, microbiota, functional food

## Abstract

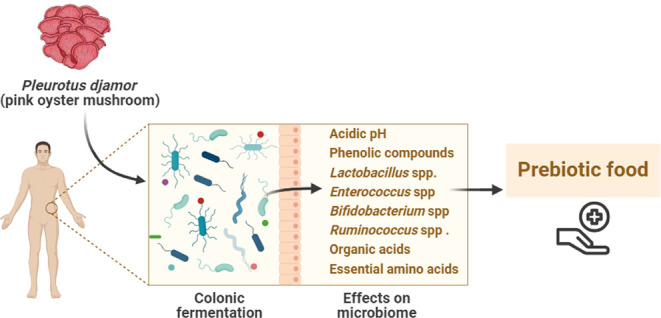

The ability of edible mushrooms to modulate the intestinal microbiota is a topic of interest. This study shows that digested *Pleurotus djamor* powder (MUS) exhibits prebiotic effects during an *in vitro* colonic fermentation. Phenolic compounds, including epicatechin (3.03 ± 1.54 mg/L), gallic acid (2.71 ± 1.54 mg/L), and quercetin 3-glucoside (2.40 ± 1.54 mg/L), were found in *P. djamor*. MUS significantly increased the relative abundance of *Lactobacillus* spp./*Enterococcus* spp. (1.12% – 4.83%), *Bifidobacterium* spp. (0.59% – 1.85%), *Ruminococcus albus/R. flavefaciens* (0.37% – 1.88%), and reduced *Clostridium histolyticum* (2.89% – 1.22%) during 48 of colonic fermentation. MUS enhanced lactic acid and short-chain fatty acid production and decreased pH levels. The ^1^H NMR analysis revealed the presence of essential amino acids, branched-chain amino acids, and other compounds that benefit human health. The results indicate the prebiotic effects of *P. djamor* on human intestinal microbiota.

## Introduction

Edible mushrooms have been used for thousands of years due to their benefits to human health and nutrition. People consume edible mushrooms worldwide, although the quantities and species consumed can vary significantly. Globally, at least 2,006 species of mushrooms are considered safe for human consumption and beneficial to human health.^1,2^ Edible mushrooms promote access to healthy and affordable food, aligning with the sustainable development goals (SDGs), including SDG 2 (Zero Hunger), SDG 3 (Good Health and Well-being), and SDG 12 (Responsible Consumption and Production).^3,4^

The production and consumption of edible mushrooms are rising, making them a promising option for a diverse and nutritious diet. Worldwide mushroom consumption continues to expand, with projections estimating it will reach 20.84 million tons by 2026. According to FAOSTAT, global mushroom production in 2021 was approximately 4 billion tons, 2-fold higher than in 2010^5^ Edible mushroom cultivation generates more than 34 billion tons annually. The sector allocates approximately 30 billion dollars to the five mushroom genera that account for 85% of global production: *>Auricularia*, *Agaricus*, *Lentinula*, *Pleurotus*,and *Flammulina*.^6^ The production data reflect the consumption patterns, as *Pleurotus* spp. is among the most consumed edible mushrooms worldwide. A recent study showed that these mushrooms are the most consumed by urban populations in Brazil.^3,7^

*Pleurotus djamor* (Rumph. ex Fr.) boedijn, also known as pink oyster, is widely distributed worldwide and is naturally found in Amazon and Atlantic Forest Brazilian biomes. Reports from traditional communities in Brazil and scientific literature confirm its edibility.^1,3,8^*P. djamor* is attractive due to its low production costs. Its lignocellulolytic exoenzymes break down tough compounds from various agronomic wastes. When integrated into a zero-waste circular economy, it promotes sustainable development in both energy and productivity.^9^ Some notable residues in *P. djamor* cultivation include banana leaves, sugar cane bagasse, coffee pulp, rice straw, and corncobs.^10,11^

Cultivating *P. djamor* is crucial for converting waste into foods with nutritional and medicinal properties. *P. djamor* is abundant in proteins, dietary fiber, vitamins, and minerals.^10,12^ This species contains various bioactive compounds, including phenolic compounds, terpenoids, ergosterol, and omega-3 fatty acids, which support health by enhancing well-being and lowering disease risk.^13−15^ Some studies have demonstrated *P. djamor* is nematicide against *Hemonchus contortus*,^16^ antibacterial against *Staphylococcus aureus* and *Escherichia coli*.^17^ Extracellular polysaccharides from *P. djamor* showed antitumor activity against Sarcoma 180 in animal models, with a tumor inhibition rate of 94%.^18^

Considering the nutritional characteristics reported in previous studies, the scarce data on functional attributes, and the biocultural relevance of *P. djamor* in improving dietary diversity,^3,4,19^ we are strategically evaluating its potential prebiotic effects. Currently, host microorganisms selectively metabolize prebiotic compounds to provide health benefits.^20^ In this context, *P. djamor* is believed to have potential prebiotic effects because other *Pleurotus* species have shown compounds linked to these impacts. Mushroom polysaccharides, particularly β-glucans, α-glucans, chitin, and phenolic compounds, are the main components attributed to these impacts.^21,22^ For instance, the β-glucan extract from *P. ostreatus* demonstrated prebiotic properties comparable to established prebiotics like fructooligosaccharide (FOS) and inulin.^23^*Lentinus edodoes* and *Pleurotus pulmonarius* showed prebiotic properties based on probiotic growth stimulation.^15^ However, the interaction between human gut microbiota and *P. djamor* is still not fully understood.

The gut microbiota is vital in maintaining intestinal homeostasis and overall health; its dysregulation can contribute to inflammatory bowel disease, diabetes, and hypertension.^24^*In vitro* studies have been widely used to simulate human intestinal conditions and assess the prebiotic efficacy of various foods and nutraceuticals, making them helpful in developing nutritional strategies.^25,26^

This study aims to explore the prebiotic properties of *P. djamor* using an *in vitro* colonic fermentation system. In a simulation of human intestinal conditions, this study assessed the impact of *P. djamor* on specific intestinal bacterial populations and metabolite production during *in vitro* colonic fermentation with a human fecal inoculum, thereby deepening the understanding of its potential health-promoting effects.

## Materials and Methods

### Collection and Characterization of *P. djamor*

*TheP. djamor* PDJR2 strain was obtained from the *Grupo de Pesquisa e Produção de Cogumelos Comestíveis* (Areia, Brazil). Under the conditions described, the mushrooms were cultivated in substrates composed of banana leaves (*Musa* spp.).^11^ The mushrooms were dried in a circulating air oven at 45 °C for 3 days and ground with a knife mill with a 16-mesh sieve. The physicochemical parameters of *P. djamor* powder were determined in a previous study,^11^ and the main physicochemical characteristics were carbohydrates 58.22 ± 1.85 g/100 g, proteins 18.77 ± 0.24 g/100 g, lipids 0.28 ± 0.08 g/100 g, dietary fiber 18.02 ± 0.05 g/100 g, and ash 4.71 ± 0.86 g/100 g. This study was registered with the Genetic Heritage Management Council (SisGen) under the A85791E code.

In this study, we analyzed the content of phenolic compounds in *P. djamor* powder before exposure to simulated gastrointestinal digestion. Two grams of *P. djamor* powder were acidified with 0.1 M HCl to reach pH 2 and mixed with 10 mL of methanol (70:30 v/v). The mixtures were incubated for 60 min at 25 °C in the dark. After centrifugation (4000 × *g* for 15 min at 24 °C), the supernatant was filtered through a regenerated cellulose membrane (pore size of 0.45 μm).^27^ The phenolic compounds (syringic acid, p-coumaric acid, gallic acid, catechin, epigallocatechin gallate, procyanidin B2, epicatechin, epicatechin gallate, procyanidin A2, and quercetin 3-glucoside) were quantified using a liquid chromatograph (1260 Infinity LC, Agilent Technologies) equipped with a Hydro-RP C18 column (150 × 4.6 mm, 4 μm). The mobile phase consisted of 4 mM/L H2SO4, and the flow rate was set to 0.7 mL/min. The system included a quaternary solvent pump (G1311C model), degasser, thermostatic column compartment (G1316A model), and automatic autosampler (G1329B model), coupled with a diode array detector (G1315D model) and a refractive index detector (G1362A model).^28^ Data were processed using OpenLAB CDS ChemStation EditionTM software (Agilent Technologies, St. Clara, CA, USA). We identified the sample peaks by comparing their retention times with the respective standards (Sigma-Aldrich, St. Louis, MO, USA).

### Simulated Gastrointestinal Digestion of *P. djamor*

First, 10 g of *P. djamor* powder was combined with 100 mL of sterilized distilled water and homogenized for 5 min. We added enzymes and specific reagents to this mixture under controlled pH, temperature (37 ± 1 °C), incubation time, and agitation intensity to simulate the various compartments of the human gastrointestinal tract, as previously described.^29^ The digestive fluids were dialyzed using a 1 kDa regenerated cellulose membrane (Spectra/Por 6, Spectrum Europe BV, Breda, Netherlands), which was prehydrated for 30 min in distilled water at 60 ± 2 °C. The predigested *P. djamor* powder was immersed in 0.01 M NaCl at 5 ± 0.5 °C. After 18 h of dialysis, the samples were frozen (−80 °C, 24 h) and then lyophilized (−55 ± 2 °C, 60 mmHg, 18–20 h) using a benchtop lyophilizer (model L-101, Brazil). The digested mushroom *P. djamor* (MUS) was then stored in a sterile, hermetically sealed glass container.

### *In Vitro* Colonic Fermentation

Following approval by the Ethics Committee for Research on Human Beings at the Federal University of Paraíba (protocol number 6.033.148), fecal samples were collected from four healthy adult volunteers (two men and two women, aged 20–39 years, with a body mass index ranging from 20.5 to 29.9 kg/m^2^). The donors were omnivores, nonsmokers, free from colonic diseases, and had not taken antibiotics or other medications that could influence the intestinal microbiota for at least three months. Additionally, they did not regularly consume concentrated prebiotics or probiotics.^30,31^ Fresh fecal samples were combined in equal proportions (1:1:1:1, w/w), diluted in sterilized phosphate-buffered saline (PBS 0.1 M; pH 7.5) at a ratio of 1:10 (w/v), and then filtered through three layers of sterilized gauze to remove large particles.^31^ ·We prepared 500 mL of sterilized fermentation medium, which had the following composition: 4.5 g of NaCl, 4.5 g of KCl, 1.5 g of NaHCO_3_, 0.69 g of MgSO_4_, 0.8 g of l-cysteine HCl, 0.5 g of K_2_HPO_4_, 0.4 g of bile salts, 0.08 g of CaCl_2_, 0.005 g of FeSO_4_, 1 mL of Tween 80, and 4 mL of a 0.025% resazurin solution in distilled water. The fermentation system consisted of 40% fermentation medium (v/v), 40% fecal inoculum (v/v), and 20% MUS (w/v), all maintained under anaerobic conditions for 48 h at 37 ± 1 °C. We used fructooligosaccharides (FOS) at 20% (w/v) as the positive control and another medium without FOS or MUS as the negative control (NC).

### Measurements of the Relative Abundance of Selected Bacteria

During *in vitro* colonic fermentation (0 and 48h), we applied fluorescence *in situ* hybridization (FISH) technique and multiparameter flow cytometry (MFC) to analyze the relative abundance of selected intestinal bacterial populations. For this purpose, SYBR Green markers (Molecular Probes, Invitrogen, Carlsbad, CA, USA) and synthesized probes labeled with Cy3 (Lab 158 specific for *Lactobacillus* spp. - *Enterococcus* spp., Bif 164 specific for *Bifidobacterium*, Rfla specific for *Ruminococcus albus/R. flavefaciens*, Bac 303 specific for *Bacteroides* spp. – *Prevotella* spp, Chis 150 specific for *Clostridium histolyticum*, and Erec 482 specific for *Eubacterium rectale* - *Clostridium coccoides*) were used. At each fermentation time, 375 μL aliquots obtained from fermentation media were fixed overnight in paraformaldehyde and hybridized with the specific fluorescent probes described previously.^32^ We used the flow cytometer (BD Accuri C6, USA) to analyze bacterial cells based on fluorescence signals detected in channels FL1 (using SYBR Green) and FL2 (which includes probes Lab 158, Bif 164, Rfla 729, Bac 303, Chis 150, and Erec 482) through the BD Accuri C6 Software. The results were expressed as the relative abundance (%) of different bacterial cells hybridized with each probe.

### Prebiotic Index

The prebiotic index was determined by calculating the relative abundance (%) of each bacterial group (FISH-MFC) and then using the following equation:^32^
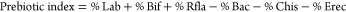
1

%Lab refers to the abundance of the bacterial population at time zero, and Lab after 24 or 48 h refers to the abundance at those respective times. The same approach was used for the other bacterial groups.

### Microbial Metabolic Activity

During *in vitro* colonic fermentation (0 and 48h), the metabolic activity analysis was performed to determine pH, sugars, lactic acid, short-chain fatty acids (SCFA), and the global metabolic profile. The pH values were determined using a potentiometric pH meter (Q400AS, Quimis, São Paulo, Brazil).^33^ The contents of glucose, fructose, maltose, rhamnose, lactic acid, acetic acid, propionic acid, and butyric acid were determined using the HPLC technique and expressed in g/L. The sample peaks were identified by comparing their retention times with the respective standards for organic acids and sugars (Sigma-Aldrich, USA).^34^

The global metabolic profile was analyzed at zero and 48 h using ^1^H nuclear magnetic resonance spectrometry (^1^HNMR). Initially, 2 mL of each sample (MUS, FOS, and NC) was diluted (1:1 v/v) in methanol and deuterated water (D2O) (9:1, v/v). The final solutions were centrifuged (MPW, 1696 × *g*, 10 min, 4 °C) and filtered with a 0.45 μm membrane filter (Millipore) for ^1^HNMR analysis. The ^1^HNMR spectra were obtained using Bruker equipment, model Avance Neo, operating at 500 MHz for ^1^HNMR and 125 MHz for 13C (Bruker, Billerica, USA). We used the following parameters: number of scans: 64; scan duration: 16; sequence Ic1pngpf2; 26 °C; receiver gain: 32; acquisition time: 3.27 s. The spectra were processed using MestReNova (MNova) software, version 5.2.3.^35^

### Statistical Analysis

The experiments were conducted in triplicate across three independent sessions, and the results were presented as mean ± standard deviation (SD). The Shapiro-Wilk normality test and Levene’s test for homogeneity of variance were applied. A mixed ANOVA with Bonferroni correction was performed to analyze the relative abundance of bacterial groups, microbial metabolic activity, and prebiotic index during colonic fermentation. Pearson’s correlation test assessed the relationship between the relative abundance of bacterial groups and microbial metabolic activity (pH and organic acids). Statistical significance was considered when *p* < 0.05, with a 95% confidence interval. All analyses were conducted using R software Version 4.1.0.

## Results

Table 1 presents the content of phenolic compounds in *P. djamor* powder. Ten compounds belonging to phenolic acids and flavonoids were detected. The phenolic compounds with the highest contents were epicatechin (3.03 ± 1.54 mg/L), followed by gallic acid (2.71 ± 1.54 mg/L), and quercetin 3-glucoside (2.40 ± 1.54 mg/L).

**Table 1:** Phenolic Compounds (Mean ± Standard Deviation; n = 3) of *Pleurotus djamor* Powder

phenolic compound	Content (mg/L)
**Phenolic Acids**	
Gallic acid	2.71 ± 1.54
Syringic acid	0.23 ± 0.76
*p*-Coumaric acid	0.17 ± 1.54
**Flavonoids**	
Catechin	1.28 ± 1.54
Procyanidin B2	1.97 ± 2.67
Epigallocatechin gallate	0.44 ± 3.45
Epicatechin	3.03 ± 1.54
Epicatechin gallate	0.34 ± 0.75
Procyanidin A2	1.37 ± 0.80
Quercetin 3-glucoside	2.40 ± 1.54

Table 2 presents the relative abundance of the bacterial populations in the fermentation medium with MUS, FOS, and NC over 48 h of *in vitro* colonic fermentation. The relative abundance of *Lactobacillus* spp./*Enterococcus* spp. increased significantly (*p* < 0.05) in the medium with FOS (2.20 ± 0.24% – 3.78 ± 0.49%) and MUS (1.12 ± 0.15% – 4.83 ± 0.32%). The relative abundance of *Lactobacillus* spp.*/Enterococcus* spp. in the medium with MUS was significantly higher than in the media with FOS and NC (*p* < 0.05). We observed an increase in the relative abundance of *Bifidobacterium* spp. in the medium containing MUS (0.59 ± 0.14% to 1.85 ± 0.37%) and FOS (1.19 ± 0.27% to 4.13 ± 0.59%) between zero and 48 h of fermentation (*p* < 0.05). The values for FOS were higher than those for MUS. At 48 h, the relative abundance of *Bifidobacterium* spp. in the medium with MUS and FOS was significantly higher than NC (1.24 ± 0.22% – 0.2 ± 0.14%) (*p* < 0.05). *R. albus/R. flavefaciens* relative abundance increased 5-fold in the medium with MUS (0.37 ± 0.16% – 1.88 ± 0.17%) from 0h to 48 h of fermentation (*p* < 0.05); there was an increase in the medium with FOS (1.58 ± 0.21% – 3.23 ± 0.26%) and decreased in NC (1.15 ± 0.16% – 0.31 ± 0.08%) (*p* < 0.05).

**Table 2:** Relative Abundance (% Mean ± Standard Deviation; n = 3) of Different Bacterial Groups in Media with Digested Mushroom *Pleurotus djamor* (MUS), Fructooligosaccharides (FOS), and Negative Control (NC; Without Fermentable Substrate) During *in Vitro* Colonic Fermentation for Different Fermentation Times^a^

Bacterial groups	Fermentation medium	0 h	24 h	48 h
*Lactobacillus* spp.*/Enterococcus* spp.	MUS	1.12 ± 0.15^Aa^	3.12 ± 0.21^Bb^	4.83 ± 0.32^Cc^
	FOS	2.20 ± 0.24^Ab^	2.64 ± 0.31^Ab^	3.78 ± 0.49^Bb^
	NC	1.83 ± 0.35^Bb^	0.29 ± 0.07^Aa^	0.51 ± 0.13^Aa^
*Bifidobacterium* spp.	MUS	0.59 ± 0.14^Aa^	1.59 ± 0.26^Ba^	1.85 ± 0.37^Ba^
	FOS	1.19 ± 0.27^Aa^	2.59 ± 0.34^Bb^	4.13 ± 0.59^Cb^
	NC	1.24 ± 0.22^Aa^	1.08 ± 0.25^Aa^	0.92 ± 0.14^Ac^
*Ruminococcus albus/R. flavefaciens*	MUS	0.37 ± 0.16^Aa^	1.25 ± 0.14^Ba^	1.88 ± 0.17^Ca^
	FOS	1.58 ± 0.21^Ab^	2.61 ± 0.32^Bb^	3.23 ± 0.26^Cb^
	NC	1.15 ± 0.16^Ab^	0.94 ± 0.27^Aa^	0.31 ± 0.08^Bc^
*Bacteroides* spp./*Prevotella* spp.	MUS	5.37 ± 0.31^Ab^	2.31 ± 0.33^Ba^	4.73 ± 0.22^Ab^
	FOS	5.14 ± 0.55^Ab^	3.43 ± 0.24^Bb^	4.54 ± 0.38^Ab^
	NC	4.56 ± 0.38^Aa^	3.88 ± 0.31^Ab^	3.23 ± 0.26^Aa^
*Clostridium histolyticum*	MUS	2.89 ± 0.17^Aa^	3.55 ± 0.28^Aa^	1.22 ± 0.38^Ba^
	FOS	4.26 ± 0.45^Cb^	3.21 ± 0.37^Ba^	2.49 ± 0.34^Ab^
	NC	3.35 ± 0.36^Aa^	3.03 ± 0.32^Aa^	2.86 ± 0.29^Ab^
*Eubacterium rectale/C. coccoides*	MUS	1.50 ± 0.35^Aa^	3.37 ± 0.28^Ba^	3.78 ± 0.17^Ba^
	FOS	3.93 ± 0.42^Ab^	2.34 ± 0.32^Bb^	2.18 ± 0.24^Bb^
	NC	3.12 ± 0.71^Ab^	3.19 ± 0.54^Aa^	2.95 ± 0.36^Aa^

a A–C: Different superscript capital letters in the same row for the same fermentation medium indicate differences (*p* < 0.05); a–c: different superscript small letters in the same column at the same time interval and bacterial group indicate difference (*p* < 0.05).

Over the 48 h of colonic fermentation, it was not observed significant changes (*p* ≥ 0.05) in the relative abundance of *Bacteroides* spp. and *Prevotella* spp. in all fermentation media. *C. histolyticum* relative abundance decreased in the medium with MUS (2.89 ± 0.17% – 1.22 ± 0.38%) and FOS (4.26 ± 0.45% – 2.49 ± 0.34%) after 48 h of fermentation (*p* < 0.05). However, only the values presented in the medium with MUS in 48h were significantly lower than NC (2.86 ± 0.29%) (*p* < 0.05). MUS induced an increase in the relative abundance of *E. rectale/C. coccoides* (1.50 ± 0.35% – 3.78 ± 0.17) at 48 h of fermentation (*p* < 0.05). In contrast, FOS (3.93 ± 0.42% – 2.18 ± 0.24%) decreased the relative abundance of *E. rectale/C. coccoides*. These values differed from the medium with MUS or NC (3.12 ± 0.71% – 2.95 ± 0.36%) (*p* < 0.05) at 48 h of fermentation.

The prebiotic indices of the fermentation medium are shown in Table 3. MUS and FOS showed a positive prebiotic index at 24 and 48 h of colonic fermentation. In contrast, NC showed a negative prebiotic index significantly different from a medium with MUS and FOS (*p* < 0.05). These differences were considerably higher at 48 h than at 24 h of colonic fermentation (*p* < 0.05).

**Table 3:** Prebiotic Index (Mean ± Standard Deviation; n = 3) Calculated for Media with Digested Mushroom *Pleurotus djamor* (MUS), Fructooligosaccharides (FOS), and Negative Control (NC; Without Fermentable Substrate) at 24 and 48 h of *in Vitro* Colonic Fermentation^a^

	Prebiotic index	
Fermentation medium	24 h	48 h
MUS	4.41 ± 0.76^Ab^	6.52 ± 1.03^Bb^
FOS	7.22 ± 1.15^Ac^	10.29 ± 1.38^Bc^
NC	-0.98 ± 0.14^Aa^	-0.49 ± 0.24^Aa^

a A–B: Different superscript capital letters in the same row for the same fermentation media indicate differences (*p* < 0.05); a–c: different superscript small letters in the same column at the same time interval indicate difference (*p* < 0.05).

The pH values, sugars, lactic acid, and SCFA during the *in vitro* colonic fermentation are shown in Table 4. MUS showed a decreased pH value at 48 h (*p* < 0.05) (6,68 ± 0,74–4,02 ± 0,28). Medium with FOS decreased the pH at 24 h (*p* < 0.05) but remained constant at 48 h (*p* ≥ 0.05). The pH in the media with FOS and MUS was lower than the control at 48h (*p* < 0.05). Regarding the sugar contents, maltose and rhamnose were detected at low levels in the medium with MUS (24 h) and differed from FOS and NC (*p* < 0.05). Unlike FOS and NC, the MUS medium presents glucose and fructose at levels below the detection limit. Lactic, acetic, propionic, and butyric acid content increased in the medium with MUS (*p* < 0.05). The medium with MUS presented the highest propionic and butyric acid contents (*p* < 0.05). At 48 h of fermentation, the propionic acid and butyric acid content increased approximately 6.5-fold and 3.7-fold compared to time zero. NC did not show lactic acid over 48 h of fermentation. Acetic, propionic, and butyric acid contents were higher in the medium with MUS and FOS than NC (*p* < 0.05).

**Table 4:** pH Values and Contents of Sugars, Lactic Acid, and Short-Chain Fatty Acids in Media with Digested Mushroom *Pleurotus djamor* (MUS), Fructooligosaccharides (FOS), and Negative Control (NC; Without Fermentable Substrate) During *in Vitro* Colonic Fermentation for Different Fermentation Times^a^

Parameters	Fermentation medium	0 h	24 h	48 h
pH	MUS	6.68 ± 0.74^Aa^	5.12 ± 0.35^Ab^	4.02 ± 0.28^Cb^
	FOS	6.58 ± 0.66^Aa^	3.48 ± 0.37^Ba^	3.14 ± 0.21^Ba^
	NC	6.62 ± 0.71^Aa^	5.04 ± 0.44^Bb^	5.01 ± 0.46^Bc^
Glucose	MUS	<LOD	<LOD	<LOD
	FOS	8.38 ± 0.96^Aa^	4.92 ± 0.38^B^	2.22 ± 0.27^C^
	NC	0.10 ± 0.17^b^	<LOD	<LOD
Fructose	MUS	<LOD	<LOD	<LOD
	FOS	7.09 ± 0.48^Ba^	7.15 ± 0.63^B^	3.60 ± 0.29^A^
	NC	0.06 ± 0.17^b^	<LOD	<LOD
Maltose	MUS	0.02 ± 0.01^Aa^	0.02 ± 0.00^Aa^	<LOD
	FOS	0.24 ± 0.02^Bb^	0.14 ± 0.02^Ab^	<LOD
	NC	<LOD	<LOD	<LOD
Rhamnose	MUS	0.06 ± 0.01^Aa^	0.05 ± 0.01^Aa^	<LOD
	FOS	<LOD	<LOD	<LOD
	NC	0.19 ± 0.02^Bb^	0.11 ± 0.01^Ab^	<LOD
Lactic acid	MUS	0.45 ± 0.07^A^	0.70 ± 0.09^Ba^	1.38 ± 0.12^Ca^
	FOS	<LOD	5.94 ± 0.36^Ab^	8.39 ± 0.77^Bb^
	NC	<LOD	<LOD	<LOD
Acetic acid	MUS	0.62 ± 0.09^Ab^	1.03 ± 0.22^Ba^	1.58 ± 0.14^Ca^
	FOS	0.30 ± 0.06^Aa^	1.36 ± 0.15^Bb^	1.81 ± 0.18^Ca^
	NC	0.23 ± 0.02^Aa^	0.30 ± 0.01^Ac^	0.45 ± 0.03^Ab^
Propionic acid	MUS	0.73 ± 0.06^Aa^	1.59 ± 0.24^Bb^	4.76 ± 0.57^Cc^
	FOS	0.77 ± 0.03^Aa^	1.48 ± 0.17^Ab^	1.66 ± 0.19^Bb^
	NC	0.98 ± 0.05^Ab^	0.30 ± 0.02^Ba^	0.23 ± 0.01^Ba^
Butyric acid	MUS	0.62 ± 0.07^Ab^	1.22 ± 0.14^Bb^	2.32 ± 0.28^Cc^
	FOS	0.31 ± 0.02^Aa^	1.13 ± 0.18^Bb^	1.29 ± 0.17^Bb^
	NC	0.31 ± 0.01^Aa^	0.73 ± 0.05^Ba^	0.85 ± 0.09^Ba^

a <LOD: below the limit of detection. A–C: Different superscript capital letters in the same row for the same fermentation medium indicate differences (*p* < 0.05); a–c: different superscript small letters in the same column at the same time interval and measured parameter indicate difference (*p* < 0.05).

**Figure 1:**
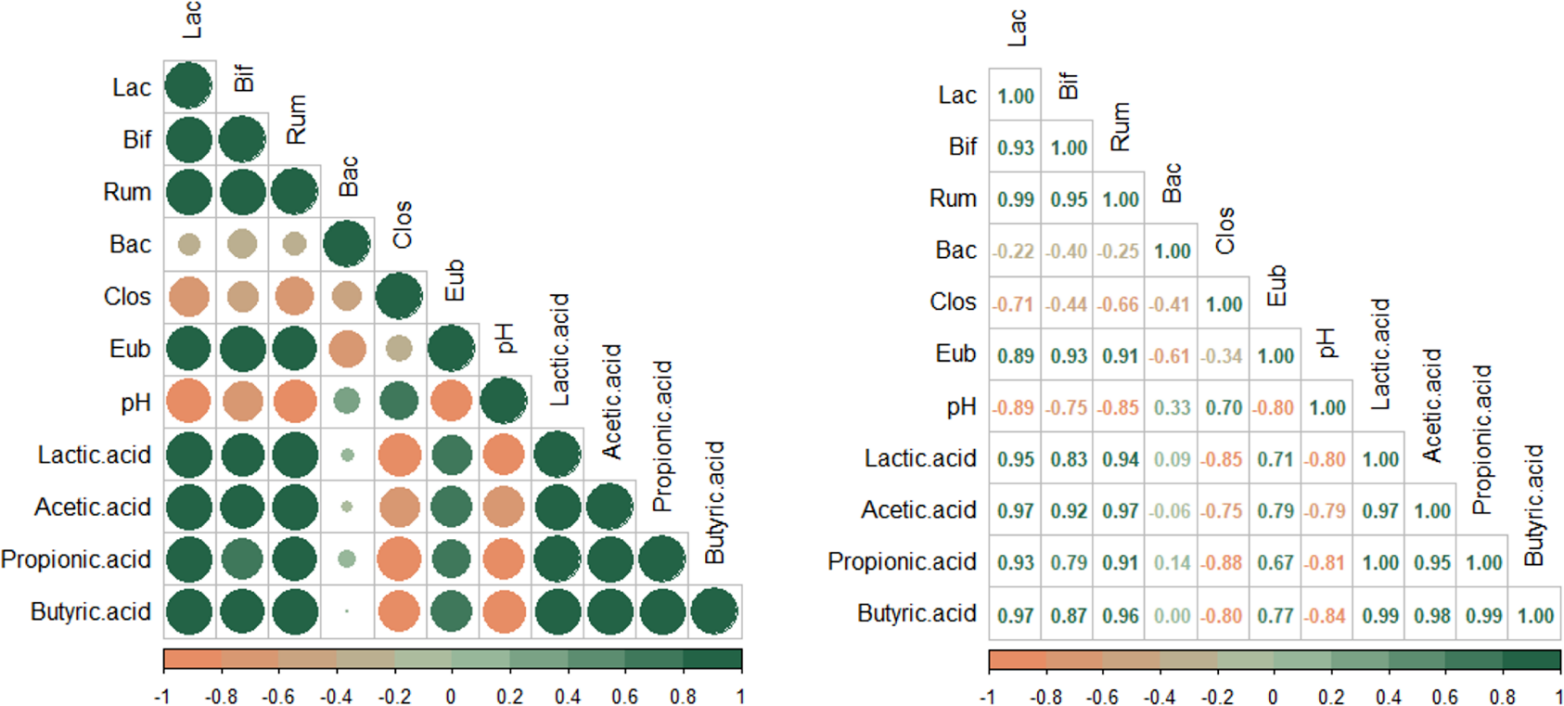
Pearson correlation matrix for data from variables measured in media with *Pleurotus djamor* (MUS) during colonic fermentation. The Pearson correlation was analyzed between the changes in the relative abundance of different bacterial groups *Lactobacillus* spp./*Enterococcus* spp. (Lac), *Bifidobacterium* spp. (Bif), *Ruminococcus albus*/*R. flavefaciens* (Rum), *Bacteroides* spp./*Prevotella* spp. (Bac), *Clostridium histolyticum* (Clos), and *E. rectale*/*C. coccoides* (Eub), pH values, and short-chain fatty acids.

The correlation between the relative abundance of bacterial groups with pH values and SCFA contents was evaluated using Pearson correlation (Figure 1). The pH values correlated negatively with the relative abundance of *Lactobacillus* spp./*Enterococcus* spp. (r: −0.89), *Bifidobacterium* spp. (r: −0.75), *R. albus*/*R. flavefaciens* (r: −0.85), and *E. rectale*/*C. coccoides* (r: −0.80). These bacterial groups showed a strong negative correlation with the contents of lactic (r: −0.80), acetic (r: −0.79), propionic (r: −0.81), and butyric acid (r: −0.84). *Bacteroides* spp./*Prevotella* spp. (r: 0.33) and *C. histolyticum* (r: 0.70) population correlated positively with pH values. *Lactobacillus* spp./*Enterococcus* spp., *Bifidobacterium* spp., *R. albus*/*R. flavefaciens*, and *Bacteroides* spp. showed a strong correlation between lactic acid and SCFA contents (R: 0.79–0.97). The strongest correlations were found between *Lactobacillus* spp./*Enterococcus* spp. and butyric (r: 0.97), acetic (r: 0.97), and lactic acid (r: 0.95); between *R. albus/R. flavefaciens* and acetic (r: 0.97) and butyric acid (r: 0.96). *C. histolyticum* negatively correlated with propionic (r: −0.88), lactic (r: −0.85), butyric (r: −0.80), and acetic acid (r: −0.75). *Lactobacillus* spp./*Enterococcus* spp., *Bifidobacterium* spp., and *R. albus/R. flavefaciens* showed a negative correlation with *Bacteroides* spp./*Prevotella* spp. and *C. histolyticum* while exhibiting a positive correlation with *E. rectale/C. coccoides*.

**Figure 2:**
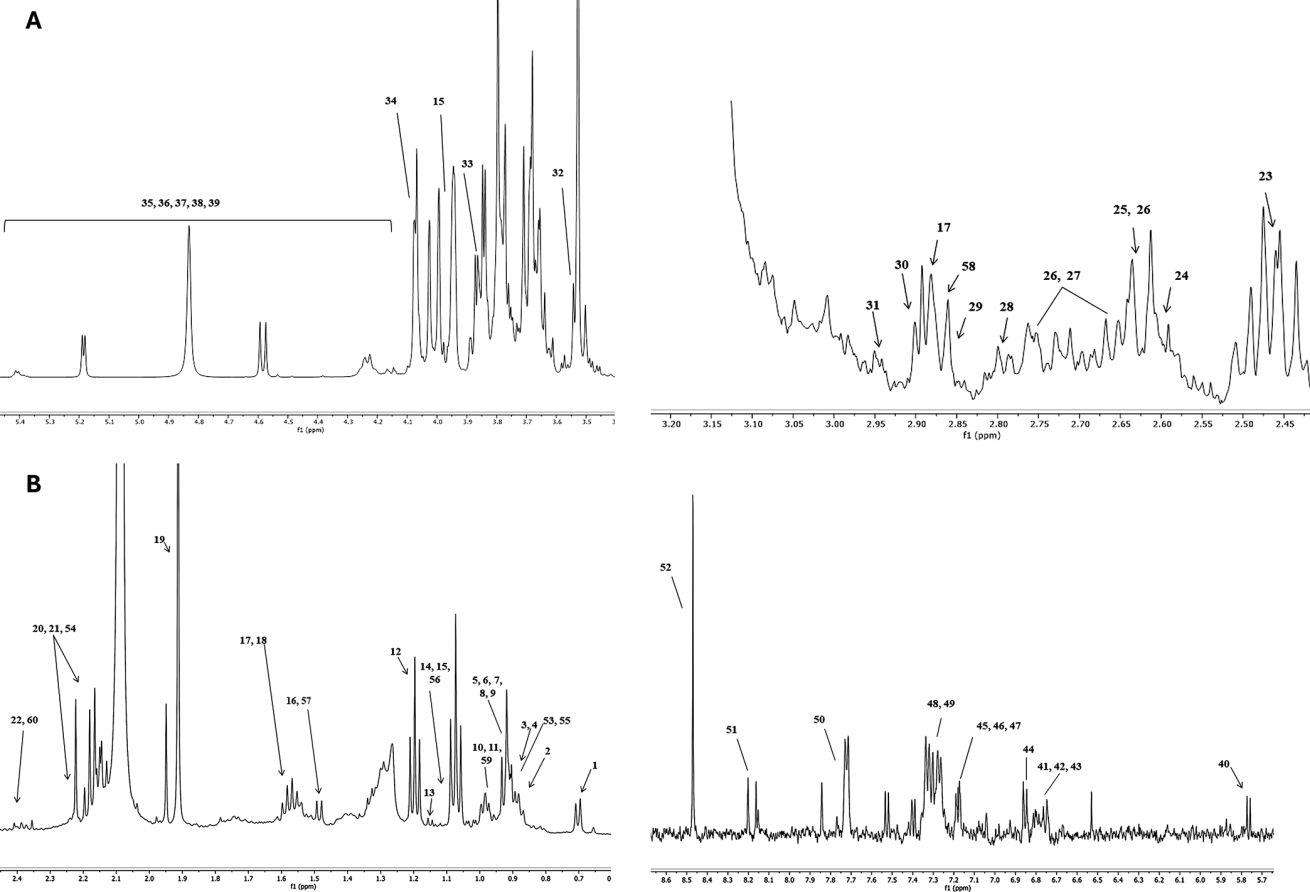
Representative ^1^H NMR spectra of the media with digested mushroom *Pleurotus djamor* (MUS) at 0 (A) and 48 h (B) of *in vitro* colonic fermentation analyzed by ^1^H NMR. 1: biliary salts; 2:2-methylbutyrate; 3: valerate; 4: n-butyrate; 5: leucine; 6: isoleucine; 7: valine; 8: propionate/propionic acid; 9: isobutyrate/butyric acid; 10:3-methyl-2-oxoisovalerate; 11:2-oxoisovalerate; 12: ethanol; 13:3-hydroxybutyrate; 14: threonine; 15: lactate/lactic acid; 16: alanine; 17: lysine; 18: ornithine; 19: acetate/acetic acid; 20: proline; 21: glutamate; 22:5-aminopentanoate; 23: succinate/succinic acid; 24: methylamine; 25: methionine; 26: citrate/citric acid; 27: aspartate; 28: asparagine; 29: trimethylamine; 30: putrescine; 31: malonate; 32: glycine; 33: fructose; 34: dihydroxyacetone; 35: α-xylose; 36: β-xylose; 37: β-glucose; 38: α-glucose; 39: D-galactose; 40: UDP-glucuronate; 41: homovanillate; 42:3-hydroxyphenylacetate; 43: p-cresol; 44: tyrosine; 45:5-aminosalicylate; 46: phenylalanine; 47: uracil; 48: N-acetyl-5-aminosalicylate; 49: phenylacetate; 50: tryptophan; 51: hypoxanthine; 52: formate/formic acid; 53: caprylate; 54: isocaproate; 55: isovalerate; 56:3-hydroxyisovalerate; 57: total lipids; 58: gamma-aminobutyric acid (GABA); 59: ketoisovalerate; 60: acetone.

The spectral profiles of the media with MUS, FOS, and NC at 0h and after 48 h of colonic fermentation revealed 60 chemical compounds (Table 5 and Figure 2). Amino acids, organic acids, and some sugars were detected at 0h and 48 h in the medium with MUS. Initially, some compounds were detected in both the MUS and FOS media; however, after 48 h of fermentation, these compounds were found only in the medium with MUS. Notable compounds included proline, glutamate, aminopentanoate, methionine, citric acid, aspartate, p-cresol, tyrosine, aminosalicylate, phenylalanine, uracil, N-acetyl-5-aminosalicylate, phenylacetate, formic acid, and isocaproate. Some compounds were identified only in the medium with MUS, such as threonine, 3-hydroxyphenylacetate, and 3-hydroxyisovalerate. Other compounds remained exclusively in the medium with MUS at 48 h of fermentation, such as acetone, 5-aminopentanoate, α-xylose, β-xylose, β-galactose, and tryptophan.

**Table 5:** Metabolites in Media with Digested Mushroom *Pleurotus djamor* (MUS), Fructooligosaccharides (FOS), and Negative Control (NC; Without Fermentable Substrate) During *in Vitro* Colonic Fermentation Analyzed by ^1^H-NMR^a^

		NC		FOS		MUS				NC		FOS		MUS	
No.	Metabolite	0 h	48 h	0 h	48 h	0 h	48 h	No.	Metabolite	0 h	48 h	0 h	48 h	0 h	48 h
1	Biliary salts	x	x	x	x	x	x	31	Malonate	x	x	x	x	x	x
2	2-Methylbutyrate	x	x	x	x	x	x	32	Glycine	x	x		x	x	x
3	Valerate	x	x	x	x	x	x	33	Fructose	x	x	x	x	x	x
4	*N*-butyrate	x	x	x	x	x	x	34	Dihydroxyacetone	x	x	x	x	x	x
5	Leucine	x		x	x	x	x	35	α-Xylose	x				x	x
6	Isoleucine	x		x	x	x	x	36	β-Xylose	x				x	x
7	Valine	x		x	x	x	x	37	β-Glucose	x				x	
8	Propionate/propionic acid	x		x	x	x	x	38	α-Glucose	x				x	
9	Isobutyrate/butyric acid	x		x	x	x	x	39	β-Galactose	x				x	x
10	3-Methyl-2-oxoisovalerate	x		x	x	x	x	40	UDP-glucuronate	x	x	x		x	
11	2-Oxoisovalerate	x		x	x	x	x	41	Homovanillate	x		x	x	x	x
12	Ethanol	x	x	x	x	x	x	42	3-Hydroxyphenylacetate					x	x
13	3-Hydroxybutyrate	x	x	x	x	x	x	43	*p*-Cresol	x	x	x		x	x
14	Threonine					x	x	44	Tyrosine	x	x	x		x	x
15	Lactate/lactic acid	x	x	x	x	x	x	45	5-Aminosalicylate	x		x		x	x
16	Alanine	x	x	x	x	x	x	46	Phenylalanine	x	x	x		x	x
17	Lysine	x	x	x	x	x	x	47	Uracil	x	x	x		x	x
18	Ornithine	x	x	x	x	x	x	48	*N*-Acetyl-5-aminosalicylate	x	x	x		x	x
19	Acetate/acetic acid	x	x	x	x	x	x	49	Phenylacetate	x	x	x		x	x
20	Proline	x	x	x		x	x	50	Tryptophan	x				x	x
21	Glutamate	x	x	x		x	x	51	Hypoxanthine	x		x		x	
22	5-Aminopentanoate	x		x		x	x	52	Formate/formic acid	x	x	x		x	x
23	Succinate/succinic acid	x			x	x	x	53	Caprylate	x		x	x	x	x
24	Methylamine	x	x	x	x	x	x	54	Isocaproate	x	x	x		x	x
25	Methionine	x		x		x	x	55	Isovalerate	x		x	x	x	x
26	Citrate/citric acid	x		x		x	x	56	3-Hydroxyisovalerate					x	x
27	Aspartate	x		x		x	x	57	Total lipids	x	x	x	x	x	x
28	Asparagine	x	x	x	x	x	x	58	γ-Aminobutyric acid (GABA)	x	x	x	x	x	x
29	Trimethylamine	x	x	x	x	x	x	59	Ketoisovalerate	x		x	x	x	x
30	Putrescine	x	x	x	x	x	x	60	Acetone	x		x		x	x

a x: Indicate the presence of the metabolite.

## Discussion

Our study analyzed the prebiotic activity of edible mushrooms *P. djamor* cultivated on banana leaves. *P. djamor* demonstrated a variety of phenolic compounds, notable content of proteins, and dietary fibers.^10^ These chemical aspects underpinned the elucidation of the prebiotic potential of *P. djamor* with an *in vitro* colonic fermentation. Ingesting a diet with natural foods, enriched foods, or dietary supplements containing prebiotic components can positively influence the microbiota. Hindgut microorganisms can selectively metabolize prebiotic components to confer health benefits.^36^

Mushrooms contain various chemical components, with phenolic compounds among the most significant groups. They have diverse therapeutic properties, including immunomodulatory, anticarcinogenic, antiviral, antioxidant, and anti-inflammatory effects.^13^ Nayak et al. (2021)^37^ reported tannins, flavonoids, terpenoids, cardiac glycosides, and saponins in *P. djamor*. The chemical profile of edible mushrooms can vary by substrate composition, culture type, and postharvest condition.^38^ A recent study showed that agro-industrial wastes (coffee pulp, rice straw, and corncobs) impacted the biological efficiency, nutritional, and functional properties of *P. djamor*.^10^ An early study reported gallic, syringic, and *p*-coumaric acid in wild *P. djamor* collected from dead tree trunks in a forest in India.^39^ Therefore, the mushrooms in this study were cultivated in banana leaves and had diverse phenolic compounds, such as epicatechin, gallic acid, and quercetin 3-glucoside (Table 1).

Wild or cultivated edible mushrooms with high phenolic compound contents generally can scavenge free radicals.^40^ Previous studies demonstrated that *P. djamor*, cultivated on banana leaves and sugar cane bagasse, had high contents of homopolysaccharides, phenolic compounds, and antioxidant activity.^11^ Preliminary chemical analyses of methanol extracts from *P. djamor* identified major bioactive components, including phenol, ascorbic acid, flavonoids, β-carotene, and lycopene, contributing to significant antioxidant activity.^41^

Additionally, phenolic compounds are recognized as modulators of gut microbiota composition. A recent systematic review of preclinical studies showed that dietary phenolics stimulate the growth of probiotic microorganisms and SCFA production.^42^ Catechin reduced the relative abundance of *Firmicutes* and the *Firmicutes*/*Bacteroidetes* while increasing *Cyanobacteria*.^43^ Quercetin reduced the relative abundance of *Proteobacteria*, *Bacteroides*, *Shigella*, and *Escherichia coli* mice with metabolic disorders.^44^ Therefore, these phenolic compounds in *P. djamor* provide access to components with prebiotic effects that benefit human health.^45^

Food rich in phenolic compounds and dietary fibers, such as *P. djamor*, are crucial in modulating intestinal microbiota and promoting gut health. The interaction between these dietary compounds and gut microbes has significant implications for human health. They can protect against oxidative stress, alleviate gut microbial dysbiosis, modulate the composition and richness of gut microbiota, protect intestinal barrier function, influence the immune system, and offer therapeutic opportunities for various diseases.^46,47^

In this study, MUS altered the relative abundance of selected bacteria and presented a positive prebiotic index (Tables 2 and 3). The prebiotic activity of *P. djamor* has been little explored. However, more consistent reports exist with other *Pleurotus* species, *Lentinula edodes*, *Hericium erinaceus*, *Ganoderma lucidum*, or their nondigestible polysaccharides. These compounds, including β-glucans, inulin, and other short-chain carbohydrates, stimulate the growth of *Bifidobacterium* and *Lactobacillus* species, considered beneficial gut microorganisms.^48^*P. djamor* grown on banana leaves and sugar cane bagasse showed a high homopolysaccharide content in ^1^HNMR analyses.^12^ These characteristics may be related to the prebiotic activity presented in this study.

*P. ostreatus* and *P. eryngii* produced glucans with prebiotic activity, supporting the growth of *Lactobacillus*, *Bifidobacterium*, and *Enterococcus* strains in a medium commonly used for lactic acid bacteria cultivation.^49^*P. citrinopileatus* polysaccharide peptides increased the populations of *Bifidobacterium*, *Lactobacillus*, *Faecalibacterium*, and *Prevotella* genera during *in vitro* colonic fermentation.^50^ In obese mice, *P. ostreatus* led to a higher relative abundance of *Oscillospira*, *Lactobacillus*, and *Bifidobacterium* while reducing *Bacteroides* and *Roseburia* populations. Consequently, the mushroom upregulated lipid metabolism, carbohydrate metabolism, and bile acid biosynthesis.^51^

Alterations in the composition of the intestinal microbiota have been linked to several chronic diseases, including obesity, dyslipidemia, type 2 diabetes, and hypertension. Most of these functional attributes are associated with the consumption of edible mushrooms.^52,53^ While there are no clinical studies on the efficacy of *P. djamor* in the literature, preclinical studies have demonstrated its pharmacological properties. For instance, dietary supplementation with *P. djamor* in healthy male mice showed no effect on anxiety-like behavior and potential for memory improvement, highlighting its safety and potential benefits.^54^

^1^H NMR is emerging as a powerful tool for analyzing metabolite profiles in various biological systems, including human fecal samples.^55,56^ MUS stimulated the production of a diverse range of microbiome components over 48 h of colonic fermentation, notably increasing branched-chain amino acids (BCAA) and SCFA, as detected by ^1^HNMR. SCFA and lactic acid are crucial in maintaining intestinal and metabolic human health by adjusting intestinal pH, promoting mucus production, and supplying energy to epithelial cells. Additionally, they play a role in modulating energy expenditure, appetite, and glucose homeostasis.^57^ SCFAs stimulate the production of TNF-α, IL-2, IL-6, and IL-10 and promote the differentiation of T cells into T regulatory cells. Additionally, they improve intestinal barrier function by promoting tight junction assembly and alleviating inflammation.^58^ These organic acids can be converted into acetyl-CoA or propionyl-CoA in the colonocytes, which enter the tricarboxylic acid cycle to produce energy. Additionally, they can activate signaling pathways through G protein-coupled receptors: GPR41, which influences leptin production in adipocytes and regulates lipid profiles,^59^ which modulates inflammation and stimulates glucagon-like peptide-1 secretion,^60^ and GPR109A reduces colonic inflammation and promotes the differentiation of regulatory T cells and IL-10-producing T cells.^61^ Butyrate, in particular, plays a crucial role in regulating tight junctions by upregulating genes encoding tight-junction proteins, thereby supporting the integrity of the intestinal barrier.^62^

BCAA (leucine, isoleucine, and valine) are essential amino acids that the gut microbiota can metabolize.^63^ In this sense, *P. djamor* can be an alternative option to bioaccess BCAA and improve the human microbiome. BCAA are crucial in maintaining intestinal health, enhancing enterocyte proliferation, improving intestinal absorption of nutrients, and strengthening immune defenses.^64^ BCAA regulate gene expression and protein synthesis associated with various signaling pathways, particularly the PI3K/AKT/mTOR pathway.^65^ These amino acids also support the intestinal barrier, regulate endocrine function, and promote the growth of probiotic bacteria in the gut.^64^

*In vitro* colonic fermentation models provide a cost-effective and reproducible alternative to *in vivo* studies. These models offer essential insights into the impact of new foods on the human colonic microbiome. However, some limitations exist, including replicating human colonic conditions.^66^ A validated *in vitro* model showed fair agreement with *in vivo* symptom reports, demonstrating its potential for predicting the gastrointestinal effects of food compounds.^67^ Despite these advancements, integrating metabolic data and addressing interindividual variations in gut microbiota remain challenges in fully understanding the influence of diet on colonic fermentation.^66^

This study demonstrated the prebiotic activity of *P. djamor* during *in vitro* colonic fermentation. This is evidenced by the positive prebiotic index and the parameters of intense metabolic activity of probiotic bacteria during *in vitro* colonic fermentation, including the production of organic acids and essential amino acids. This discovery is a significant advancement, inspiring further research with well-structured preclinical and randomized, double-blind clinical studies, including placebo controls. These studies are necessary to ensure safety and efficacy in humans and to better understand the mechanisms of action, especially regarding cardiometabolic diseases influenced by the modulation of the intestinal microbiota. Furthermore, our data provide relevant information on the potential use of mushrooms as functional foods, demonstrating that they can be classified as a prebiotic food in conventional form or through processes that facilitate access to nutrients and bioactive compounds, such as the formulation of nutraceuticals and dietary supplements.
